# Transcript Profiling Reveals Auxin and Cytokinin Signaling Pathways and Transcription Regulation during *In Vitro* Organogenesis of Ramie (*Boehmeria nivea* L. Gaud)

**DOI:** 10.1371/journal.pone.0113768

**Published:** 2014-11-21

**Authors:** Xing Huang, Jie Chen, Yaning Bao, Lijun Liu, Hui Jiang, Xia An, Lunjin Dai, Bo Wang, Dingxiang Peng

**Affiliations:** College of Plant Science and Technology, Huazhong Agricultural University, #1 Shizishan Street, Hongshan District, Wuhan 430070, Hubei Province, China; Chinese Academy of Sciences, China

## Abstract

*In vitro* organogenesis, one of the most common pathways leading to *in vitro* plant regeneration, is widely used in biotechnology and the fundamental study of plant biology. Although previous studies have constructed a complex regulatory network model for Arabidopsis *in vitro* organogenesis, no related study has been reported in ramie. To generate more complete observations of transcriptome content and dynamics during ramie *in vitro* organogenesis, we constructed a reference transcriptome library and ten digital gene expression (DGE) libraries for illumina sequencing. Approximately 111.34 million clean reads were obtained for transcriptome and the DGE libraries generated between 13.5 and 18.8 million clean reads. *De novo* assembly produced 43,222 unigenes and a total of 5,760 differentially expressed genes (DEGs) were filtered. Searching against the Kyoto Encyclopedia of Genes and Genomes Pathway database, 26 auxin related and 11 cytokinin related DEGs were selected for qRT-PCR validation of two ramie cultivars, which had high (Huazhu No. 5) or extremely low (Dazhuhuangbaima) shoot regeneration abilities. The results revealed differing regulation patterns of auxin and cytokinin in different genotypes. Here we report the first genome-wide gene expression profiling of *in vitro* organogenesis in ramie and provide an overview of transcription and phytohormone regulation during the process. Furthermore, the auxin and cytokinin related genes have distinct expression patterns in two ramie cultivars with high or extremely low shoot regeneration ability, which has given us a better understanding of the *in vitro* organogenesis mechanism. This result will provide a foundation for future phytohormone research and lead to improvements of the ramie regeneration system.

## Introduction

*In vitro* organogenesis, one of the most common pathways leading to *in vitro* plant regeneration, is used in biotechnology and the fundamental study of plant biology [1], [2]. Organogenesis is a multistep process consisting of callus formation, adventitious organ formation and micropropagation using axillary or apical meristem containing tissues [3]. The process is largely controlled by the balance between auxin and cytokinin [4]. Morphological, biochemical and molecular methods have been used to investigate the mechanisms behind phytohormone signaling and gene regulation during *in vitro* organogenesis in several plants, such as Arabidopsis [5], poplar [3] and almond [6]. Previous studies have constructed a complex regulatory network model for *in vitro* organogenesis, particularly for Arabidopsis [2], [7], [8]. The results from our experiments will build on these previous studies.

Ramie (*Boehmeria nivea* L. Gaud) is an important natural fiber crop of the Urticaceae family and is well-known for its smooth, long fibers with excellent tensile strength. Therefore, ramie is widely cultivated in China, India and other Southeast Asian and Pacific Rim countries. In China, ramie is the second most important fiber crop after cotton, in terms of cultivated area and fiber production. Thus far, ramie studies have mainly focused on fiber development [9], [10], fiber processing [11]–[13], and environmental stresses [14]–[17], with the aim of improving fiber quality and production. In contrast, there have been no reports on the mechanisms controlling *in vitro* organogenesis, even though regeneration systems and genetic transformation have been successfully applied to ramie [18]–[22]. Organogenesis systems are widely applied because the explant and culture conditions are simpler than somatic embryogenesis techniques [2]. The organogenesis systems in many plants share similarities with Arabidopsis and the two step regeneration protocol for Arabidopsis is commonly applied to many plants [3], [7], [23], [24]. However, we used a slightly different organogenesis system, i.e. explants were incubated on one single medium that contained both auxin and cytokinin [18].

In previous studies, a number of genes that were activated or differentially expressed during *in vitro* organogenesis were cloned and validated by various molecular techniques [25]–[29]. Suppression subtractive hybridization (SSH) was then used to identify differentially expressed genes on a small scale [6] and microarray techniques were used to give a global overview of the large number of genes involved in *in vitro* organogenesis, which indicated that different plants probably had distinct gene expression patterns [3], [5]. Recently, RNA-seq has been used in plant biology, mainly utilizing the 454 platform [30], the Illumina [31] and the ABI SOLiD [32] systems. Several studies on shoot development have already been reported [33], [34]. For plants without any genomic information, RNA-seq has proved to be a fast and efficient method for obtaining large numbers of functional genes [35]–[38]. RNA-seq has already been applied in fiber development and drought stress studies of ramie and a series of key genes have been classified [10], [14]. In general, RNA-seq is an effective method for identifying key regulators that controls ramie *in vitro* organogenesis.

To generate more complete observations of transcriptome content and dynamics during ramie *in vitro* organogenesis, we used a reference transcriptome for mixed samples and digital gene expression (DGE) for single samples. This was undertaken using Illumina sequencing. To our knowledge, this is the first genome-wide gene expression profiling of *in vitro* organogenesis in ramie. The data will serve as a foundational resource for further studies into ramie development, phytohormone signaling and improvements to the organogenesis system.

## Materials and Methods

### Plant materials and sampling

**Figure 1:**
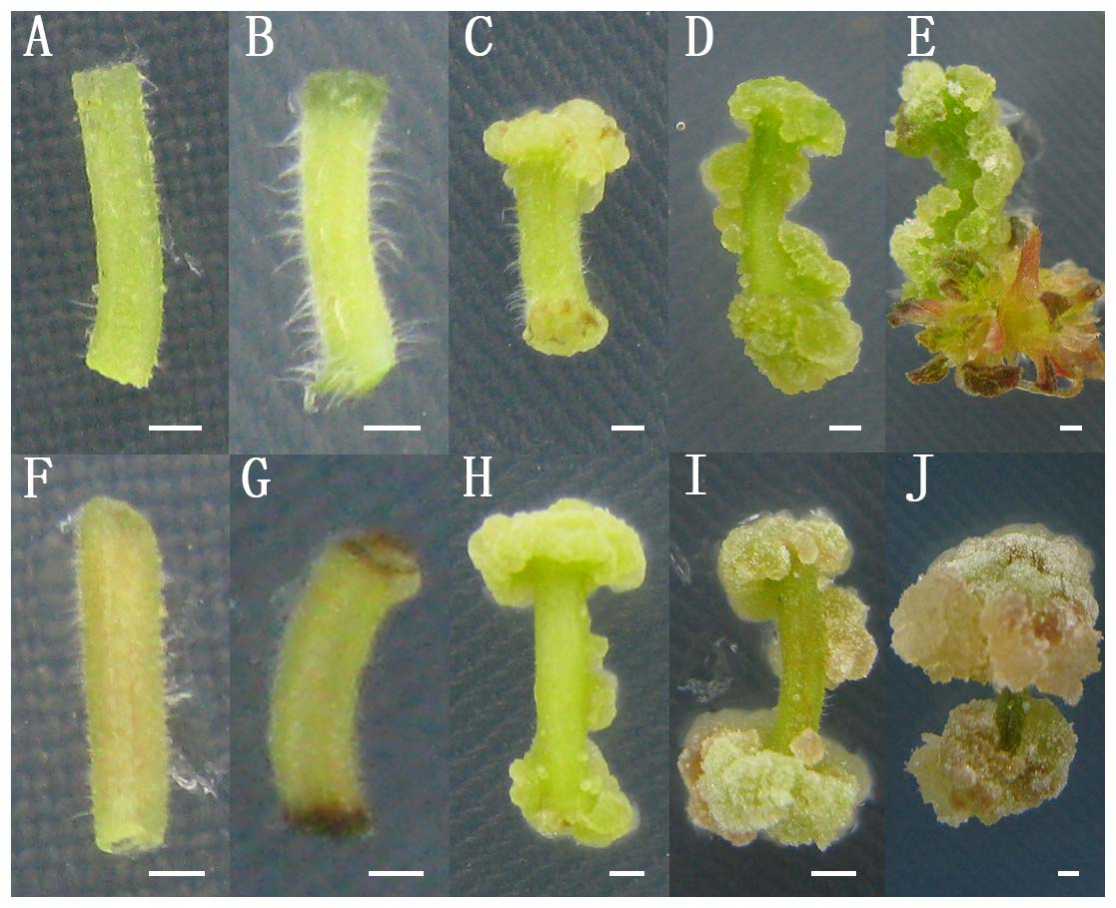
Morphological observation of H5 and DZ at different time points during in vitro organogenesis. Petiole explants from H5 (A–E) and DZ (F–J) were sampled for RNA extraction at five sequential time points. They were incubated on MS medium containing TDZ (0.25 mg/L) and NAA (0.06 mg/L) for 0 (A, F), 4 (B, G), 14 (C, H), 28 (D, I) and 35 (E, J) days.

Two ramie cultivars, Huazhu NO. 5 (H5) and Dazhuhuangbaima (DZ), with approximately 80% and 0.1% regeneration frequencies, respectively, were used in this study. The plants were propagated *in vitro* according to published protocols [18]. Petiole segments (3–6 mm in length) from *in vitro* micropropagated plants were cut and incubated on MS medium, containing 0.25 mg/L TDZ (Sigama) and 0.06 mg/L NAA (Sigma), in a culture room under cool white fluorescent light with a 16/8 h (light/dark) cycle at 25±2°C during the day and 20±2°C at night. RNAs were extracted from two batches (representing two replications) that had been grown under the same growth conditions, but two weeks apart, in November 2013. The samples were collected at 0, 4, 14, 28 and 35 days (Figure 1). Approximately 12–18 petiole explants from the same plate were pooled for RNA extraction.

### RNA extraction, library preparation and sequencing

Ramie samples at the different growth stages of H5 were collected and the total RNA was isolated from each sample using a Tiangen RNA prep Pure Plant Kit (Tiangen Biomart, Beijing). Twenty micrograms of total RNA from each sample was sent to Novogene Bioinformatics Technology Co. Ltd (Beijing), where the libraries were constructed, and sequenced using the Illumina HiSeq 2000 platform. RNA quality and quantity were determined by a Nano Photometer spectrophotometer (IMPLEN, CA, USA), a Qubit RNA Assay Kit in a Qubit 2.0 Flurometer (Life Technologies, CA, USA) and a Nano 6000 Assay Kit that was part of the Agilent Bioanalyzer 2100 system (Agilent Technologies, CA, USA). A total of 10 µg RNA, 1 µg from each of the ten samples, was used as the input material for the transcriptome library and 3 µg RNA per sample was used for the DGE library. Briefly, the mRNA was purified by poly-T oligo-attached magnetic beads and fragmented by divalent cations under elevated temperature in NEB Next First Strand Synthesis Reaction Buffer (5×). Random hexamer primer and M-MuLV Reverse Transcriptase (RNase H) were used for first strand cDNA synthesis. Second strand cDNA synthesis was subsequently performed using DNA Polymerase I and RNase H. These double-stranded cDNA fragments were end-repaired by adding a single ‘A’ base and ligation of adapters. The adaptor modified fragments were selected by gel purification and amplified, through PCR, to create the final cDNA library. Transcriptome sequencing was carried out on an Illumina HiSeq 2000 platform that generated 100 bp paired-end raw reads, while DGE sequencing generated 100 bp single-end raw reads.

### De-novo assembly and functional annotation

The raw reads of our transcriptome (1 record) and DGE (10 records) data were deposited in the Sequence Read Archive (SRA, http://www.ncbi.nlm.nih.gov/Traces/sra/) under accession number SRP041146. After removing the adaptor sequences, ambiguous ‘N’ nucleotides (where the ‘N’ ratio was more than 10%), low quality sequences (where the quality score was less than 5) and the remaining clean reads were none reference genome assembled by Trinity [39].

The non-redundant sequences were subjected to public databases so that their functional annotations could be identified. These were the NCBI (http://www.ncbi.nlm.nih.gov/) non-redundant protein (Nr) and non-redundant nucleotide (Nt) databases, the Protein Family database (Pfam) (http://pfam.sanger.ac.uk/), Swiss-Prot (http://www.ebi.ac.uk/uniprot/), Gene Ontology (GO) (http://www.geneontology.org/), the euKaryoticOrtholog Groups database (KOG) (http://www.ncbi.nlm.nih.gov/COG/) and the Kyoto Encyclopedia of Genes and Genomes (KEGG) (http://www.genome.jp/kegg/). Comparisons between the Nr, Nt and Swiss-Prot databases were carried out by BlastX with an E-value cut-off of 10^−10^, while COG and KEGG classifications had a cut off E-value of 10^−5^.

### Analysis of DGE tags and bioinformatics

Raw reads generated by single-end sequencing were also submitted to the NCBI Short Read Archive (SRA) database (http://www.ncbi.nlm.nih.gov/sra). After trimming, the clean reads were mapped back onto the assembled transcriptome and the read count for each gene was derived from the mapping results obtained by RSEM, an user-friendly software package for quantifying gene and isoform abundances from single-end or paired-end RNA-Seq data [40]. All read counts were normalized to reads per kilo bases per million mapped reads (RPKM) [41]. DESeq was used to determine differential expressions and was based on the negative binomial distribution [42]. Genes with an adjusted P value<0.05 were accepted as being differentially expressed.

### The qRT-PCR analysis of auxin and cytokinin related genes

The RNA extracted from the different H5 and DZ development stages were used for qRT-PCR. Reverse cDNA for each sample was generated using the GoScript Reverse Transcription System (Promega, USA), according to the manufacturer's instructions. An optical 96-well plate iQ5 multicolor real time PCR system (Bio-RAD, USA) was used for the qRT-PCR. Each reaction contained 1 µL of cDNA template, 10 nM gene-specific primers, 10 µL of iTaq Universal SYBR Green Supermix (Bio-RAD, USA) and 7 µL of ddH_2_O in a final volume of 20 µL. The glyceraldehyde-3-phosphate-dehydrogenase (GAPDH) gene was selected as the endogenous control [43]. Gene-specific primers (Table S1) were designed, according to the cDNA sequences, using Primer 3 (http://primer3.ut.ee/), which were synthesized commercially by Sunny Biotech, Shanghai. The thermal cycle used was as follows: 95°C for 5 min, followed by 40 cycles of 95°C for 15 s and 60°C for 30 s. Following amplification, a dissociation stage was carried out to detect any complex products. The qRT-PCR was performed in triplicate for each sample. Relative expression levels were calculated as described previously [44].

## Results

### Callus and shoot development during regeneration

Petiole explants, which have a simpler physiology and structure than leaf explants and no axillary buds compared to stem explants, were selected for culturing on regeneration medium containing TDZ and NAA. They were incubated for 4 days, after which the explants had a deep color at their ends (Figure 1B, G). Cali began to form at the two ends after 7 days and their size continued to increase (Figure 1C, H). Adventitious shoot buds were observed after 30 days incubation and grew to 5 mm in length after 35 days (Figure 1E). Based on morphological observation, we set five time points at 0 (W0), 4 (W1), 14 (W2), 28 (W3) and 35 (W4) days for sampling.

Two ramie cultivars, H5 and DZ, were used for the preliminary regeneration experiment. Adventitious shoot buds regenerated from most of the H5 petiole explants (Figure 1E), whereas few developed on the DZ petiole explants after 35 days of culturing (Figure 1J). The two cultivars therefore had distinctly different regeneration frequencies. As a result, H5 petiole explants were used for RNA-seq analysis and petiole explants from both cultivars were employed in the qRT-PCR validation process.

### Generating a ramie reference transcriptome by Illumina sequencing

To obtain a global and comprehensive overview of the ramie transcriptome, RNA was extracted from the different petiole explants of H5 (incubated for 0, 4, 14, 28 and 35 days) and then mixed together. Approximately 117.28 million raw reads were generated by Illumina paired-end sequencing and 111.34 million clean reads (94.94% of raw reads) were retained for further analysis after a stringent quality filtering process. *De novo* assembly of clean reads by Trinity software [39] produced 43,222 unigenes. The length distribution of the unigenes is shown in Figure S1. There were 22,844 unigenes (52.85%) with lengths ranging from 200 to 500 bp, 7,474 unigenes (17.29%) with lengths varying from 501 bp to 1,000 bp, 7,358 unigenes (17.02%) with lengths ranging from 1,001 bp to 2,000 bp and 5,546 unigenes (12.83%) with lengths that were longer than 2,000 bp.

All the unigenes were compared to the NCBI non-redundant protein sequences (Nr) and the NCBI nucleotide sequences (Nt) using a cut-off E-value of 10^−5^. As a result, 19,275 (44.59%) unigenes were annotated in the NCBI Nr database and 10,395 (24.05%) unigenes were annotated in the Nt database. We also conducted comparisons using the Pfam database [45], which showed that 35.43% (15,314) of the unigenes demonstrated similarity to known genes. Moreover, we also identified 14,832 (34.31%) unigenes that had an ortholog in the Swiss-Prot database (Table S2).

**Figure 2:**
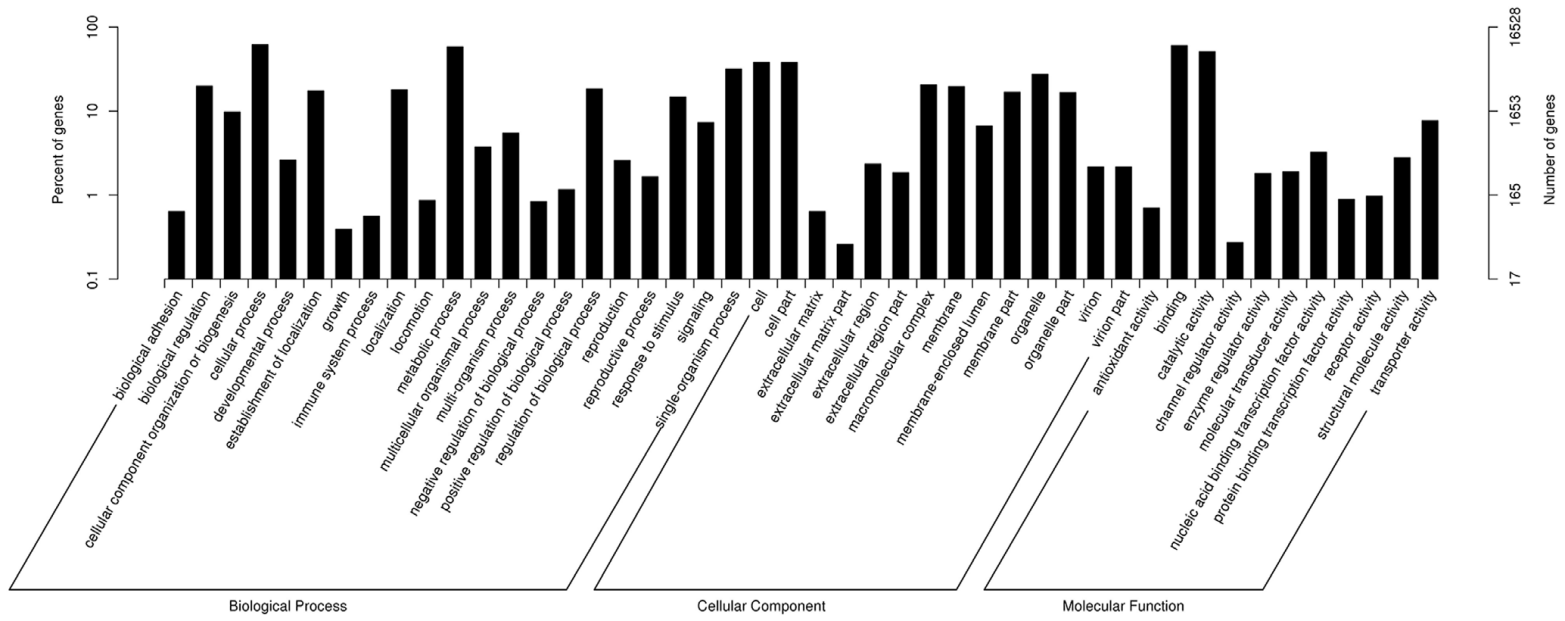
Gene Ontology classifications for the assembled unigenes. The results are classified into three main categories: biological process, cellular component and molecular function.

**Figure 3:**
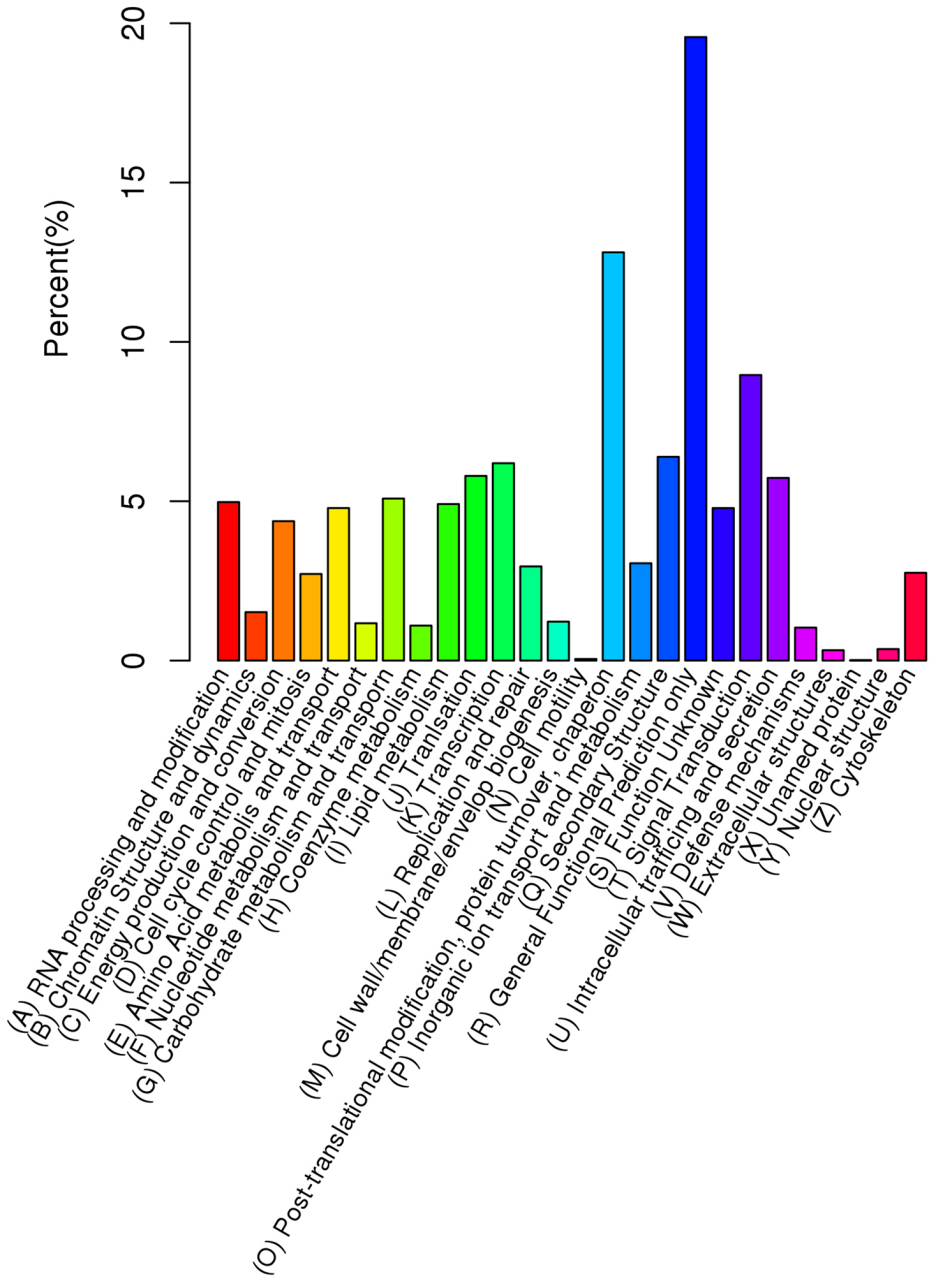
Distribution of the assembled unigenes across the KOG categories.

According to Gene Ontology (GO) [46], 16,528 (38.23%) unigene sequences could be categorized into three major categories (biological process, cellular component and molecular function) and 55 subcategories. Six subcategories, ‘cellular process’, ‘binding’, ‘metabolic process’, ‘catalytic activity’, ‘cell’ and ‘cell part’, were dominant clusters in the GO classification (Figure 2; Table S2).The euKaryotic Ortholog Groups program (KOG) [47] matched and grouped 8,024 unigenes into 26 functional classes. The clusters for ‘general function prediction only’ (1,570), ‘post-translational modification’, ‘protein turnover’, ‘chaperon’ (1,028) and ‘signal transduction’ (719) were the three largest groups and represented 19.57%, 12.81% and 8.96%, respectively (Figure 3; Table S2). Furthermore, pathway analysis was carried out using the Kyoto Encyclopedia of Genes and Genomes (KEGG) [48], This showed that 5,700 unigenes were functionally assigned to 31 KEGG pathways. The most represented pathways were ‘metabolic pathways’ (2,649 members) and ‘genetic information processing’ (1,293 members) (Figure S2). These annotations provide a valuable resource for investigating specific processes, functions and pathways during ramie *in vitro* organogenesis.

### Global analysis of differential gene expression during in vitro organogenesis

To generate digital expression signatures for ramie *in vitro* organogenesis, we sequenced two libraries as replicates for each developmental stage (petiole explants of H5 were incubated for 0, 4, 14, 28 and 35 days, designated as W0, W1, W2, W3 and W4). In total, the DGE libraries generated between 13.8 and 19.2 million raw reads. After removing insignificant reads, the total number of clean reads per library ranged from 13.5 to 18.8 million. The clean reads per library were mapped to the reference transcriptome database using RSEM software [40] and represented between 93.1% and 94.2% of the overall total (Table S3).

Gene expression levels were determined by calculating the number of clean reads mapped to the reference database for each gene (read count) and then normalizing to reads per kilobases per million mapped reads (RPKM) [41]. To examine the reliability of expression data between replicates, the Pearson correlation coefficient was calculated by SPSS with transformation of log10 (RPKM+1). The correlations between samples of each development stage were all over 0.8, which indicated the reliability of replicates (Figure S3, R2 = 0.830 of W0, R2 = 0.945 of W1, R2 = 0.858 of W2, R2 = 0.967 of W3 and R2 = 0.963 of W4). All the read counts in each library were analyzed by DESeq [42], which identified the differentially expressed genes (DEGs), based on a negative binomial distribution. A total of 5,760 DEGs were filtered with a p_adj_<0.05 (Table S4).

**Figure 4:**
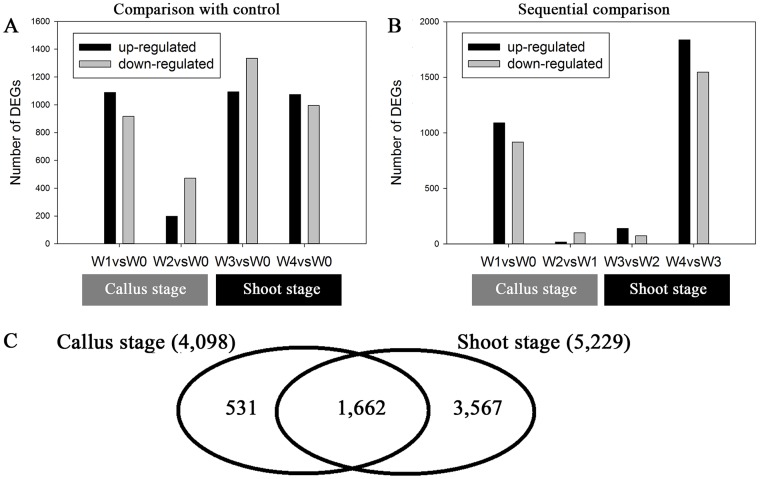
Histogram and Venn diagram of DEGs during in vitro organogenesis. Differential expression was calculated by comparison with the pre-induction stage (control) (A) and with the prior sampling point (B). The DEGs were identified using DESeq. The Venn diagram shows specifically or commonly expressed DEGs during the callus and shoot stages (C).

The number of genes that were up- or down-regulated at the different developmental stages is shown in Figure 4A, B. There were 2,193 and 5,229 DEGs during the callus and shoot stages, respectively (Figure 4C). Of these, 28.85% of the DEGs were present during both developmental stages. There were 531 specifically expressed genes during the callus stage and 1,662 during the shoot stage, which suggested the presence of distinct spatial transcriptional profiles.

**Figure 5:**
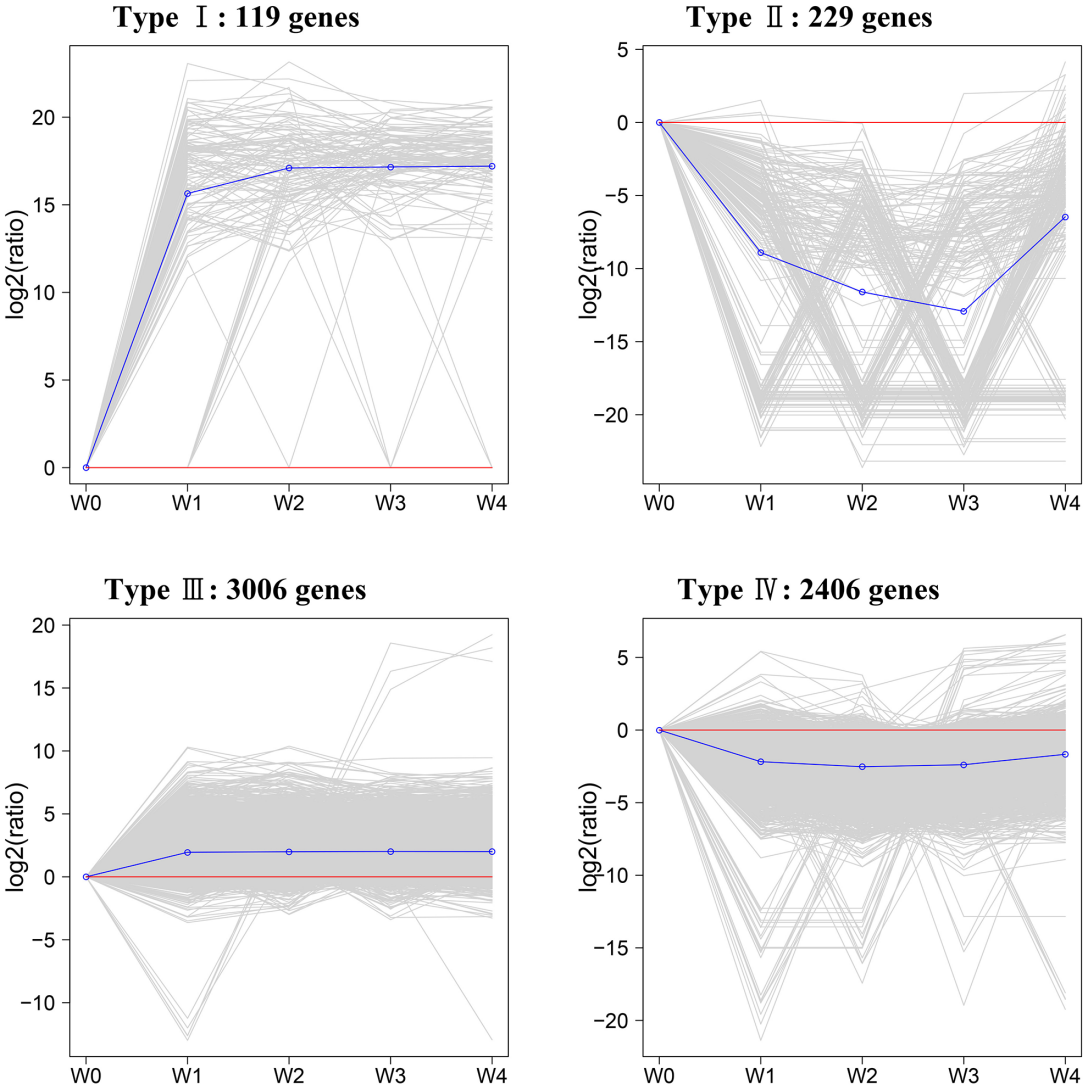
Differentially expressed genes during in vitro organogenesis were clustered by the k-Means method, which is based on their expression modulation. The relative expression level was obtained after taking equation and logarithmic transformations of RPKM.

The 5,760 genes were classified into four expression types (Figure 5) using the k-Means method, which is based on their expression modulation. Type I genes were positively modulated during early callus induction and less positively during late callus induction. Type II genes were up-regulated during early shoot induction and then down-regulated during late shoot induction. Type III genes were down-regulated during early callus induction and had relatively low expression levels throughout the whole process and type IV genes were down-regulated during callus induction and up-regulated during shoot induction, although their overall expression levels were relatively low. The expression data for each gene type are shown in Table S5.

### Transcription Factor mRNA present during in vitro organogenesis

From the 5,760 DEGs in H5 libraries, we identified 248 transcription factors (TFs) mRNAs in 36 published TF families in the Database of Arabidopsis Transcription Factors (DATF) [49]. The TF mRNAs were classified by Nr annotation and are shown in Table S6. Zinc finger, MYB and AP2/ERF family TFs were the three largest families and represented two fifths of all TFs (Table 1). The bHLH family TFs and others, such as HB, WRKY, NAC, bZIP and ARF, accounted for 3.6% to 8.4%, respectively, of the TFs, which was far more than the other families. In total, more TFs were detected at the shoot stage than at the callus stage and more down-regulated TFs were expressed over the whole development process than up-regulated TFs (Table 1). All major TF families were represented in the mRNA population at each developmental stage and several TF families were specifically expressed during particular developmental periods.

**Table 1:** Regulated transcription factors during in vitro organogenesis.

Gene family	No.	Percentage	W1 vs W0		W2 vs W0		W3 vs W0		W4 vs W0	
			Up	Down	Up	Down	Up	Down	Up	Down
zinc finger	48	19.4%	6	18	2	11	13	25	14	20
C2H2*	12	4.8%	1	5	1	1	2	7	2	6
TAZ*	11	4.4%	1	4		3	3	6	3	4
Dof*	10	4.0%	3	1		2	2	5	2	3
CO-like*	10	4.0%	1	4	1	4	6	4	6	4
C3H*	2	0.8%		2				2		2
PHD*	2	0.8%		1					1	
GATA*	1	0.4%		1		1		1		1
MYB	32	12.9%	13	8	4	7	13	8	16	8
AP2/ERF	26	10.5%	2	10	2	11	7	17	7	13
bHLH	21	8.5%	3	8		6	7	13	7	9
HB	16	6.5%		3		2	3	13	2	6
WRKY	12	4.8%		5		2	2	9	3	5
NAC	11	4.4%	1	5		5	3	6	2	6
bZIP	9	3.6%	3	1			6	2	5	1
GRAS	9	3.6%	1	6		2		8	1	3
ARF	8	3.2%	1	3		2	1	5	1	4
MADS	8	3.2%		2		1	4	2	5	2
AUX/IAA	6	2.4%		2		1		6		2
LBD	5	2.0%	3		3	1	3	2	3	1
ABI3	4	1.6%	2	1			1		1	
LUG	3	1.2%	1				2	1		
SET	3	1.2%	3				2		1	
HSF	3	1.2%	1	1	1		2	1	2	1
CAMTA	3	1.2%		1				3		3
GRF	3	1.2%							3	
Trihelix	3	1.2%					1	2	1	1
TCP	2	0.8%		1			1		1	
JUMONJI	2	0.8%		2				2		2
E2F	2	0.8%	2							
HMG	2	0.8%	1				1		2	
LIM	2	0.8%		1				1	1	1
CCAAT	1	0.4%							1	
TLP	1	0.4%	1				1		1	
FHA	1	0.4%	1							
PLATZ	1	0.4%								1
Nin-like	1	0.4%		1						
Total	248	100%	45	79	12	51	73	126	80	89

*Subgroup of zinc finger family.

### Auxin and cytokinin signalling pathways during in vitro organogenesis

**Figure 6:**
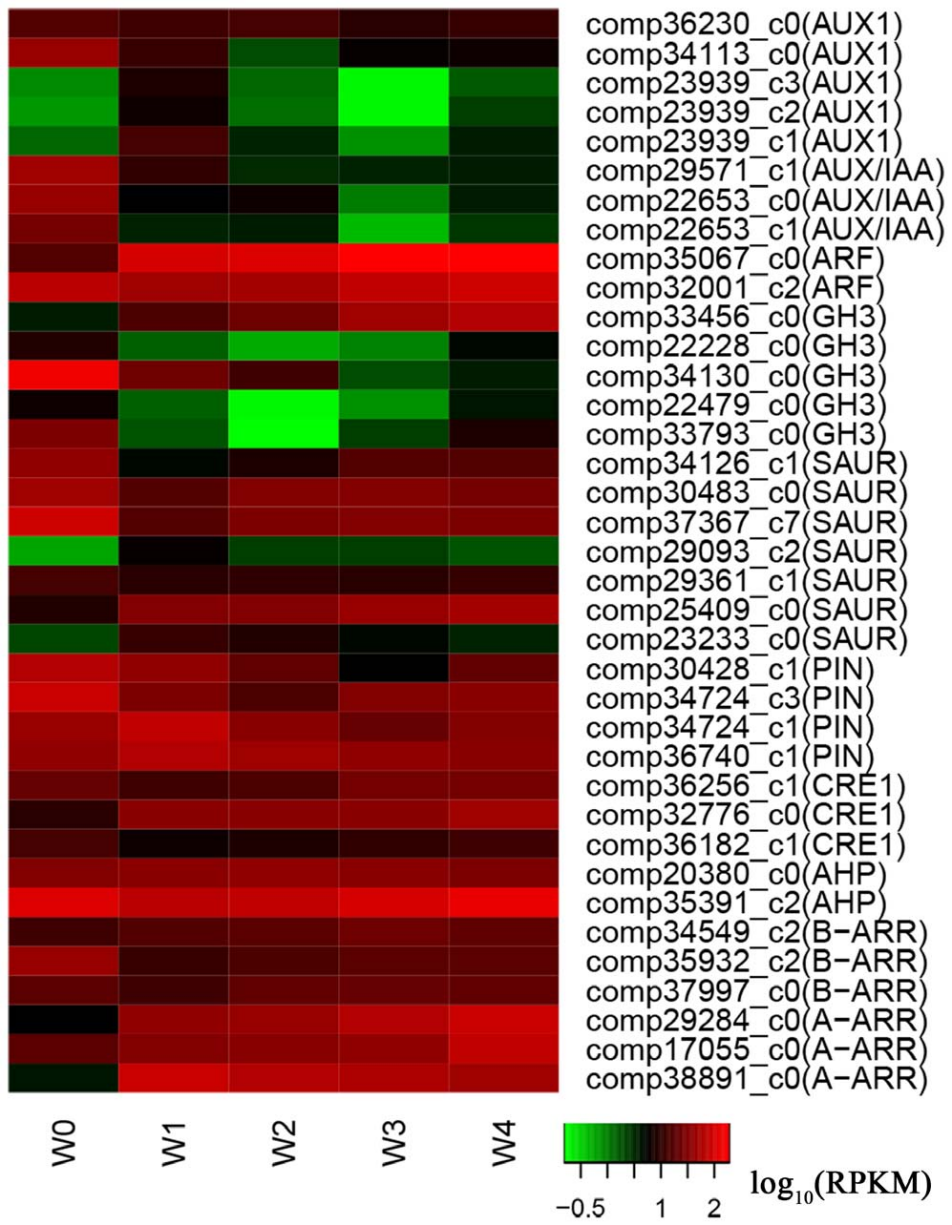
Differentially expressed genes related to auxin and cytokinin signaling. The relative expression levels were obtained by RNA-Seq after taking logarithmic transformations of RPKM.

In this study, 26 genes related to auxin signaling pathway, were differentially expressed during *in vitro* organogenesis (Table S7). Based on the KEGG results, these genes were identified as being related to auxin influx carrier (AUX1, 5 transcripts), auxin efflux carrier (PIN, 4 transcripts), auxin response protein (Aux/IAA, 3 transcripts), auxin response factor (ARF, 2 transcripts), auxin responsive GH3 gene (GH3, 5 transcripts) and small up-regulated RNAs (SAUR, 7 transcripts). Two AUX1 transcripts (comp36230_c0 and comp34113_c0), three Aux/IAA transcripts (comp29571_c1, comp22653_c0 and comp22653_c1), one ARF transcript (comp32001_c2) and two PIN transcripts (comp30428_c1 and comp34724_c3) were down-regulated during callus induction. The other three AUX1 transcripts (comp23939_c3, comp23939_c2 and comp23939_c1) and two PIN transcripts (comp34724_c1 and comp36740_c1) were up-regulated and then down-regulated during callus induction. All the transcripts were up-regulated during shoot induction. The other ARF transcript was up-regulated throughout the whole development process. In addition, we found that GH3 and SAUR transcripts had multiple expression patterns (Figure 6), which indicated that auxin signaling regulation was complex.

In cytokinin signaling pathway, we identified 11 transcripts that were classified as cytokinin receptors (CRE1, 3 transcripts), histidine-containing phosphotransferproteins (AHP, 2 transcripts) and two-component response regulators (ARR-A, 3 transcripts; ARR-B, 3 transcripts), according to the KEGG results. Among these, one CRE1 transcript (comp32776_c0) and two A-ARR transcripts (comp29284_c0 and comp17055_c0) were up-regulated throughout the development process. One AHP transcript (comp20380_c0), one B-ARR transcript (comp34549_c2) and one A-ARR transcript (comp38891_c0) were up-regulated and then down-regulated, while the other five transcripts were down-regulated and then up-regulated (Figure 6).

**Figure 7:**
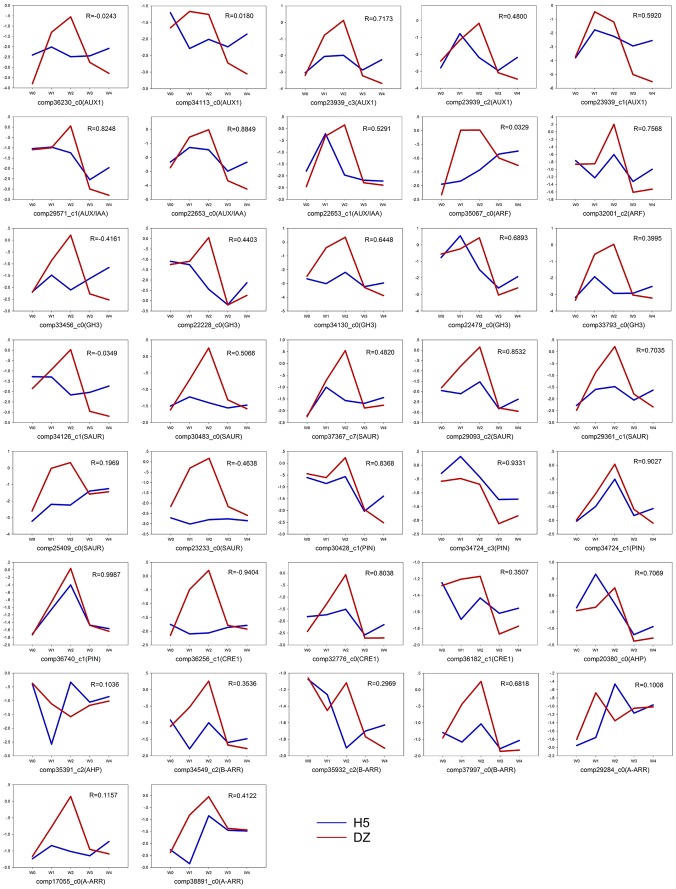
Expression pattern and correlations for auxin and cytokinin related genes in two ramie cultivars, H5 and DZ. The relative expression level was obtained by qRT-PCR after logarithmic transformation of the data. The correlation coefficient (R) was calculated by SPSS.

For qRT-PCR validation, both cultivars (H5 and DZ) were used for culturing and sampling at the five time points. The 37 differentially expressed transcripts related to phytohormone signaling were tested in H5 and DZ. The results showed that most auxin and cytokinin transcripts were up- or down-regulated at lower levels in H5 than in DZ. The correlation coefficient was calculated by SPSS to assess the relationship between the two cultivars. The results revealed that only eight transcripts shared highly similar expression patterns in both cultivars (R>0.8). The expressions of the remaining transcripts showed lower or negative correlations between H5 and DZ (Figure 7).

### Comparisons of qRT-PCR with DGE data of ramie and microarray data of Arabidopsis

**Figure 8:**
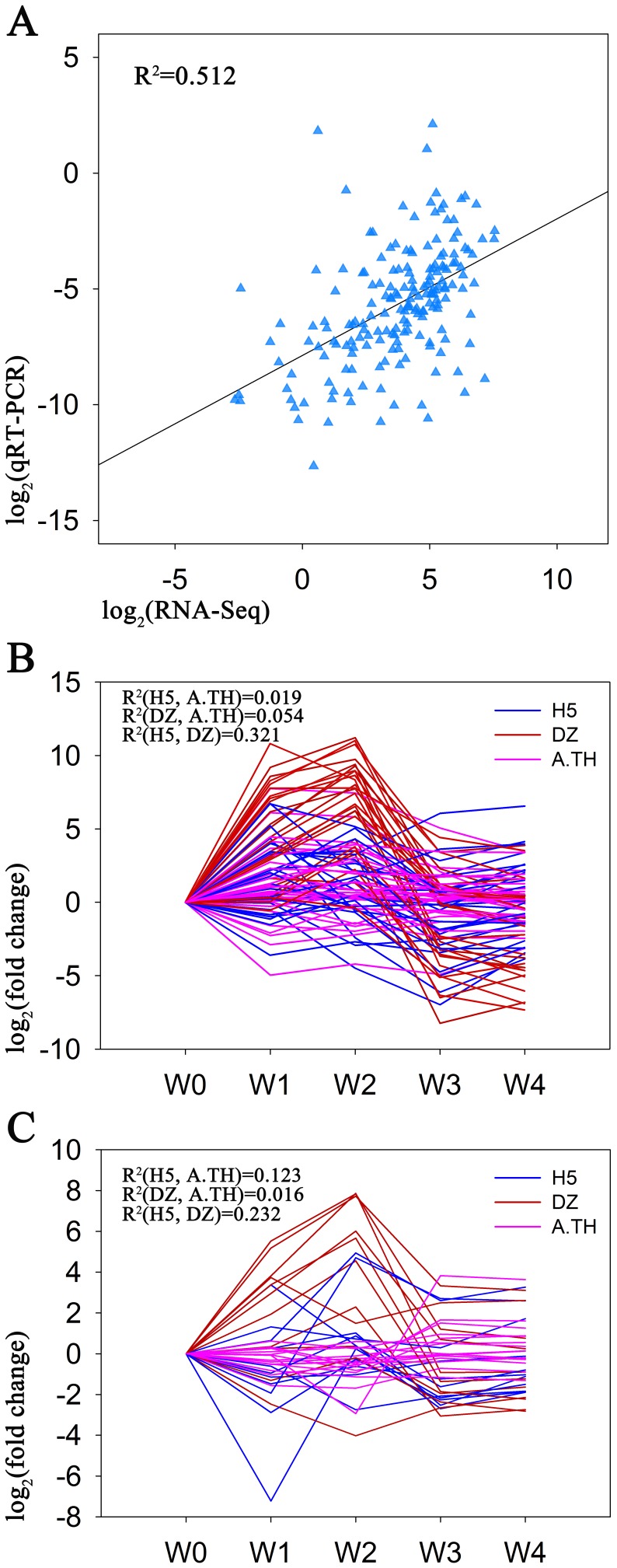
Comparisons of qRT-PCR with DGE data of ramie and microarray data of Arabidopsis. Comparison of expression profiles of 37 phytohormone related genes by qRT-PCR and RNA-Seq in ramie cultivar H5 (A). Comparisons of expression profiles of auxin (B) and cytokinin (C) related genes in H5 (blue), DZ (red) and Arabidopsis (A.TH, purple). The expression data of Arabidopsis genes was obtained from a published article [5].

To validate the expression profiles from RNA-Seq, qRT-PCR was performed on the 37 phytohormone signaling related genes in H5. The Pearson correlation coefficient was calculated to assess the correlation between different platforms by SPSS. The validation of the 37 phytohormone signaling related genes showed a moderate correlation (Figure 8A, R^2^ = 0.512, correlation was significant at the 0.01 level). The result suggested the applicability of RNA-Seq to ramie transcriptome analysis and confirmed that it is an accurate and reliable method to find DEGs during shoot organogenesis.

Furthermore, we compared expression pattern of the 37 genes in H5 and DZ with expression data of Arabidopsis homologs (Table S7) during shoot organogenesis reported in previous study [5]. By calculating correlation coefficient in SPSS, we found that auxin signaling related genes shared a slightly higher relationship than those of cytokinin in H5 and DZ (Figure 8B, C). Compared with Arabidopsis homologs, auxin signaling related genes showed relatively low correlations both in H5 and DZ (R^2^ = 0.019 and 0.054, respectively). Whereas, cytokinin signaling related genes showed relatively higher correlation in H5 than in DZ with Arabidopsis homologs (R^2^ = 0.123 and 0.016, respectively). Auxin related genes between H5 and DZ also showed higher correlations than cytokinin related genes (R^2^ = 0.321 and 0.232, respectively).These results indicated a more distinct expression pattern of cytokinin signaling related genes than auxin in the two different ramie genotypes.

## Discussion

### Evaluation of the reference transcriptome

Until now, no genomic data has been available for ramie plants, which has greatly restricted molecular studies of the crop. The development of RNA-seq should lead to more transcript profiling analysis. In our study, 111.34 million clean reads were generated by short-reads (Illumina) sequencing and was nearly twice as large as the previous pool [10]. Full length transcriptome assembly was carried out by Trinity software, which has an outstanding none reference genome assembly performance [39]. As a result, nearly 30% of the unigenes were more than 1 kbp long and 50% were over 500 bp long (Figure S1), which demonstrated that the assembly was no less effective than the 454 GS platform [50] in capturing a large portion of transcriptome. More unigenes longer than 1,000 bp were obtained in this study compared to a previous study [10]. However, 52.8% of the unigenes were shorter than 500 bp and only one fifth of them were annotated (Figure S1), and more longer-length unigenes were annotated in this study. Less than 1% of the unigenes that were longer than 2 kb did not match with any of the homologous sequences. Clearly, the length and completeness of the assembled unigenes affected annotation rates. Moreover, a lack of homologous sequences in public databases may indicate that ramie contains a number of unique unigenes.

### Analysis of DEGs

It is well known that complex cellular and molecular networks regulate *de novo* shoot organogenesis and a large number of functional genes have been isolated in model plants [2], [7], [8]. Very few studies have investigated *de novo* shoot organogenesis in ramie. Therefore, we used a DGE method to obtain in-depth knowledge of organogenesis using the highly efficient regeneration cultivar, ‘Huazhu No. 5’. Five morphological development stages, petiole explants, early callus induction, callus, early shoot induction and shoot formation, were designated for DGE analysis. According to the DGE results, 2,193 unigenes were differentially expressed during callus induction and 1,149 of them were up-regulated and 1,044 down-regulated. This was much less than the 5,038 and 3,429, respectively, in Arabidopsis or the 5,681 and 5,959 in poplar, respectively [3]. A total of 3,567 shoot specifically expressed unigenes occurred in ramie compared to just 478 in Arabidopsis [5]. These results indicated that different genome-wide regulation was occurring between the two species.

There were several possible reasons for this, of which different technologies and species might be two main factors. Recently, several reports have shown that both microarray and RNA-seq have advantages, depending on the application. For example, RNA-seq is a better choice for transcript discovery and isoform identification [51]–[54]. We obtained a large amount of callus and shoot related genes that were annotated in the public databases, which makes them available for further studies on cellular and molecular networks. Different species tend to have characteristic gene expression patterns. For example, poplar shared less than one fourth homology with Arabidopsis during callus and shoot development [3]. Similarly, we found 466 differentially expressed unigenes during callus induction and there were no matched homologies in the public databases. Some of these genes might represent new *in vitro* organogenesis transcripts that had not occurred in the model plants, which means that they need further study.

### Transcription regulation of in vitro organogenesis in ramie

Molecular biology methods have revealed that transcription regulation is complex and have classified key TFs involved in *in vitro* organogenesis [2], [3], [5], [7], [8]. This has provided valuable references for related studies on ramie. In this investigation, 248 TF mRNAs that differentially expressed during *in vitro* organogenesis were associated with callus and shoot induction processes. Zinc finger family proteins have been shown to be involved in development [55]; OsLSD1, a rice zinc finger protein, regulates callus differentiation [56]; Arabidopsis MINI ZINC FINGER1 (MIF1) and MIF3 genes induce shoot meristems [57] and HANABA TARANU (han) is a GATA transcription factor that regulates shoot apical meristems and flower development [29]. Zinc finger TFs in ramie also showed complex expression patterns (Table 1), which indicated that they were involved in many different functions during *in vitro* organogenesis. In Arabidopsis, MYB proteins are key regulators controlling development and metabolism [58]. Ramie MYB TFs had diversity expressions throughout the entire development process. A series of molecular biology studies on shoot meristem development revealed that complex transcription regulation networks existed and that these contained AP2/ERF, bHLH, HB, WRKY, NAC, bZIP, GRAS and MADS transcription factors [25], [26], [28], [59]–[65]. Moreover, the ARF, AUX/IAA and LBD families also have roles in auxin-mediated signaling [66]. Several reported ABI3 and CCAAT TFs are related to embryogenesis, which may bring breakthroughs to related studies of ramie [67]–[69]. The TFs in these families had different expression patterns during ramie *in vitro* organogenesis.

The TF mRNAs in this study belonged to 36 TF families. They had all been detected in poplar and most showed similar expression profiles [3]. Exceptionally, AP2/ERF TFs in ramie showed distinct expression differences compared to poplar. More up-regulated TFs were identified in poplar, but more down-regulated TFs were found in ramie. Previous studies identified the functions of two Arabidopsis AP2/ERF TFs: ESR1 overexpression triggers shoot regeneration and ectopic expression of BBM induces spontaneous somatic embryo formation [70].The up-regulated AP2/ERF TFs in ramie may have similar functions and this needs to be investigated in future studies. The differences in down-regulated TFs need further investigations in order to validate the physiological functions and interrelations between TFs and other genes during *in vitro* organogenesis in ramie.

### Auxin and cytokinin signaling pathways during in vitro organogenesis of ramie

The auxin and cytokinin signaling pathways have been identified previously and both phytohormones serve as key regulators during plant development [71]–[75]. There is complex crosstalk between auxin and cytokinin, which triggers root and shoot organogenesis [76]. We identified homologous transcripts in five families of auxin signal transduction factors and four families of cytokinin signal transduction factors, according to KEGG. Interestingly, no transport inhibitor response 1 (TIR1) ortholog was classified in the KEGG results, although it is an auxin receptor [77]. It might be that TIR1 expression is extremely low during *in vitro* organogenesis compared to model plants and therefore it was not detected. However, we did obtain AUX1 and PIN transcripts, which are major auxin influx and efflux carriers [78]. By co-regulation of AUX1 and PIN, the polar transport of auxin forms ectopic maxima, which triggers callus formation [2], [71]. In this study, five AUX1 and four PIN transcripts were involved in two opposite expression modulations, respectively (Figure 6). This suggests that there is a complex auxin flux during *in vitro* organogenesis of ramie. However, more studies are needed to reveal how transporters regulate auxin distribution. Under IAA treatment, Aux/IAA proteins are up-regulated and repress ARF proteins [79]. Besides, GH3 proteins regulate auxin conjugation to decrease free auxin level [80]. Although the cultures incubated on medium containing auxin, we found that Aux/IAA transcripts showed opposite expression patterns, so did ARF and GH3 transcripts (Figure 6). The phenomena might be caused by auxin polarity transport, thus most regions of callus contained lower auxin level than zones with actively dividing cells, such as meristems [2], [81].

Cytokinin regulates plant development through the classic two component regulatory systems [82]. Our KEGG results showed that a similar system existed in ramie. Previous studies have revealed that A-ARR were up-regulated under cytokinin stimulus [83], [84], which would repress WUS expression to maintain normal shoot apical development [85]. Moreover, CRE1 upstream regulates A-ARR together with AHP and B-ARR [73], [84]. In this study, we found that most genes of the four families were significantly up-regulated during shoot stage (Figure 6), suggesting their direct roles in cytokinin signaling. These results will provide a foundation for further cytokinin studies in ramie.

The auxin and cytokinin machineries are highly conserved [86]–[88]. Therefore we conducted qRT-PCR validations of the two ramie cultivars, which had high (H5) or extremely low (DZ) shoot regeneration abilities. Previous studies suggested that genotype was a key factor affecting *in vitro* shoot organogenesis [89]–[92]. Indeed, the two cultivars, H5 and DZ, had distinct phenotypes during shoot regeneration under the same phytohormone conditions. By qRT-PCR analysis, we found that only seven auxin related transcripts and one cytokinin related transcript shared highly similar expression patterns in H5 and DZ (Figure 7, correlations were more than 0.8). Furthermore, we compared the integral expression pattern of the 37 genes in H5 and DZ separately with Arabidopsis homologs. We found that most genes in DZ were higher up-regulated during callus stage and down-regulated during shoot stage than H5 and Arabidopsis (Figure 8B, C). This suggests that DZ cells might be more sensitive to auxin, which is also shown in other plants that auxin gradients lead to different phenotypes [90], [92]. Interestingly, cytokinin related genes in H5 showed a relatively high correlation with Arabidopsis than DZ. Therefore, we speculate the regulation of cytokinin related genes might contribute to the different phenotypes. The differing regulation of auxin and cytokinin might be caused by the different original climates. It has been shown that hormone levels and responses are modulated by environmental cues and these can change plant growth [73], [93]. DZ was first cultivated in Sichuan province, which has a more humid climate than east Hubei province where H5 was firstly planted. The original growth habits of the two cultivars might cause different response regulations under stress. The differences in auxin and cytokinin regulation between the two cultivars will be further studied in order to evaluate their shoot regeneration abilities and to reveal the evolution of ramie cultivars.

## Conclusions

Bioinformatic methods are highly effective on detecting modulations in gene expression during development. In our study, 43,222 unigenes were obtained using Illumina paired-end sequencing technology. We also conducted the first genome-wide gene expression profiling of *in vitro* organogenesis in ramie and have provided an overview of transcription and phytohormone regulation during *in vitro* organogenesis. Furthermore, 37 auxin and cytokinin signaling related transcripts were identified in our research and their expression patterns were analyzed in two ramie cultivars with high or extremely low shoot regeneration ability. The distinct expression patterns have given us a better understanding of the *in vitro* organogenesis mechanism, which will provide a foundation for future phytohormone research and lead to improvements in the ramie regeneration system.

## Supporting Information

Figure S1 **Histogram of unigene length distributions and the proportion of sequences annotated in at least one database.**
(JPG)

Figure S2 **KEGG classification of non-redundant unigenes.**
(JPG)

Figure S3 **The Pearson correlation coefficients between replicates were calculated by SPSS with transformation of log10(RPKM+1).**
(TIF)

Table S1 **Primers of ramie GAPDH gene (as endogenous control) and 37 auxin and cytokinin genes used for qRT-PCR.**
(XLSX)

Table S2 **The annotated unigene number in public databases.**
(XLSX)

Table S3 **Statistics of DGE sequencing.**
(XLSX)

Table S4 **The unigene information, gene expression level, BLASTx against nr protein sequence with E-value≤10−10 and the GO term for 5,076 differentially expressed genes.**
(XLSX)

Table S5 **Expression data (log2-ratio) of each type genes.**
(XLSX)

Table S6 **List and categories of putative transcription factors.**
(XLSX)

Table S7 **The unigene information and expression levels of auxin and cytokinin related genes differentially expressed during the process.**
(XLSX)
