# Supplementary figures and images for: Transcript Profiling Reveals Auxin and Cytokinin Signaling Pathways and Transcription Regulation during In Vitro Organogenesis of Ramie (Boehmeria nivea L. Gaud)
Source: PLoS One. 2014 Nov 21;9(11):e113768. doi: 10.1371/journal.pone.0113768 (PMC4240604; doi:10.1371/journal.pone.0113768)

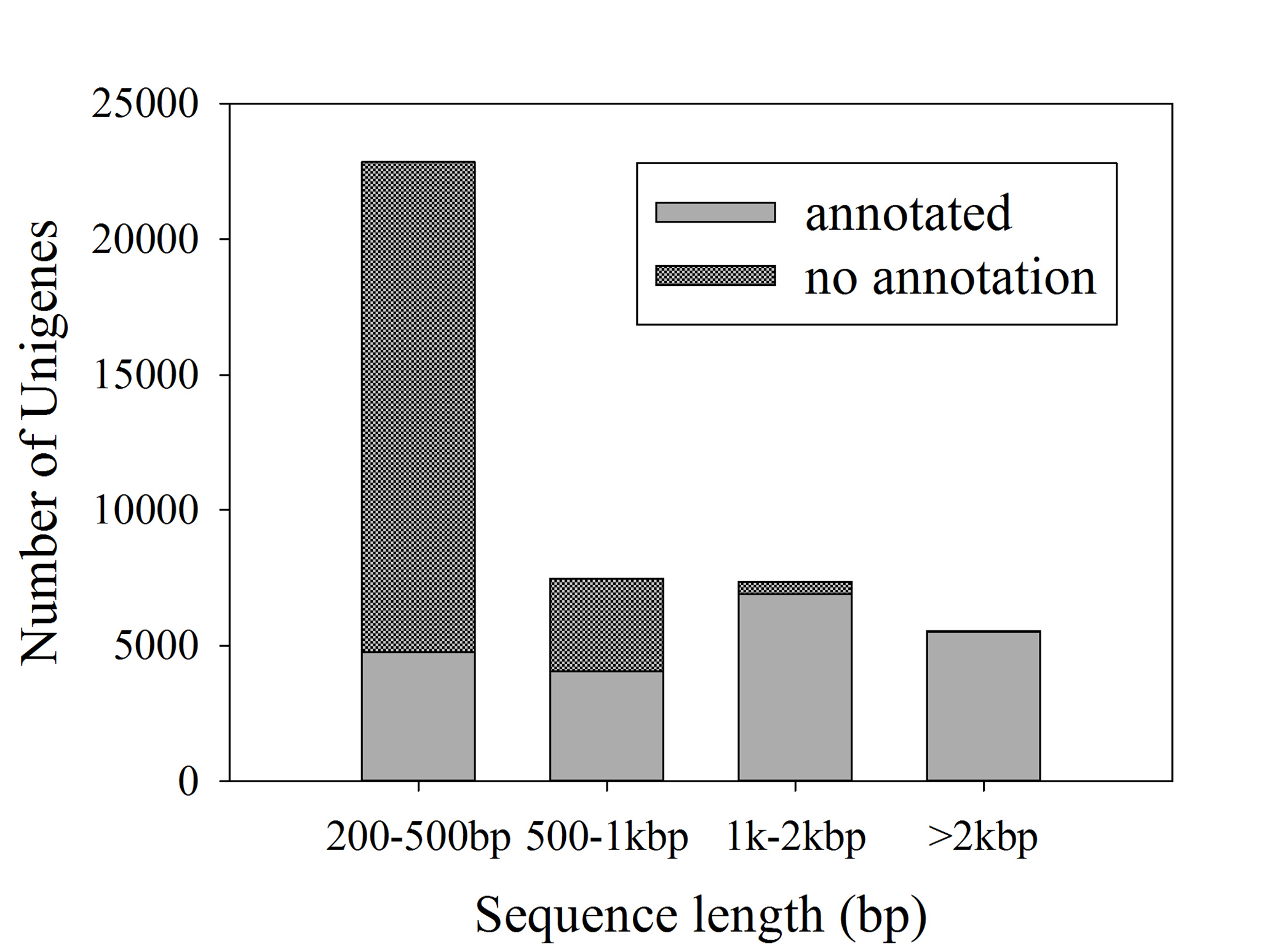

Supplement: Figure S1 — Histogram of unigene length distributions and the proportion of sequences annotated in at least one database. (JPG) [file pone.0113768.s001.jpg]

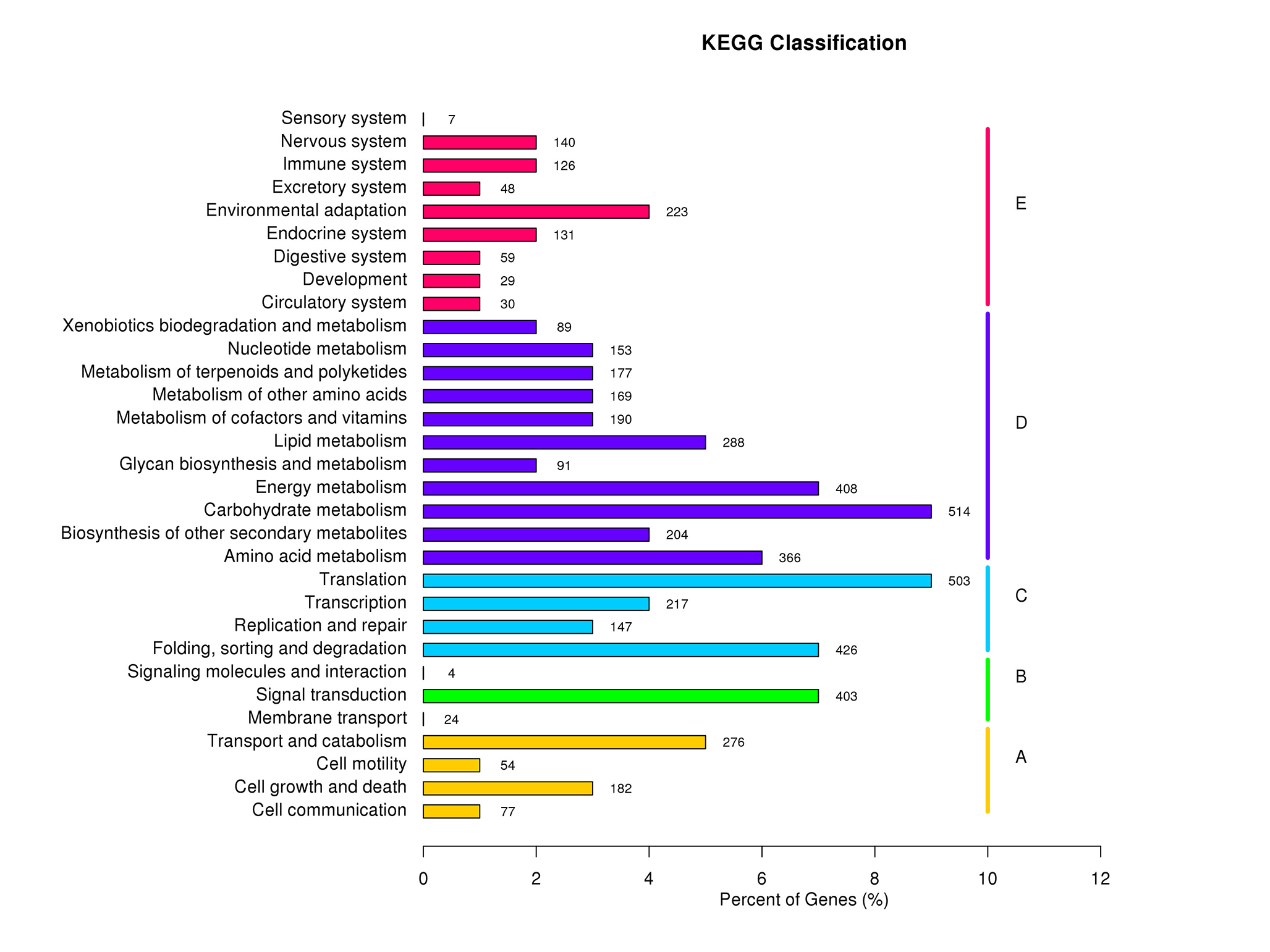

Supplement: Figure S2 — KEGG classification of non-redundant unigenes. (JPG) [file pone.0113768.s002.jpg]

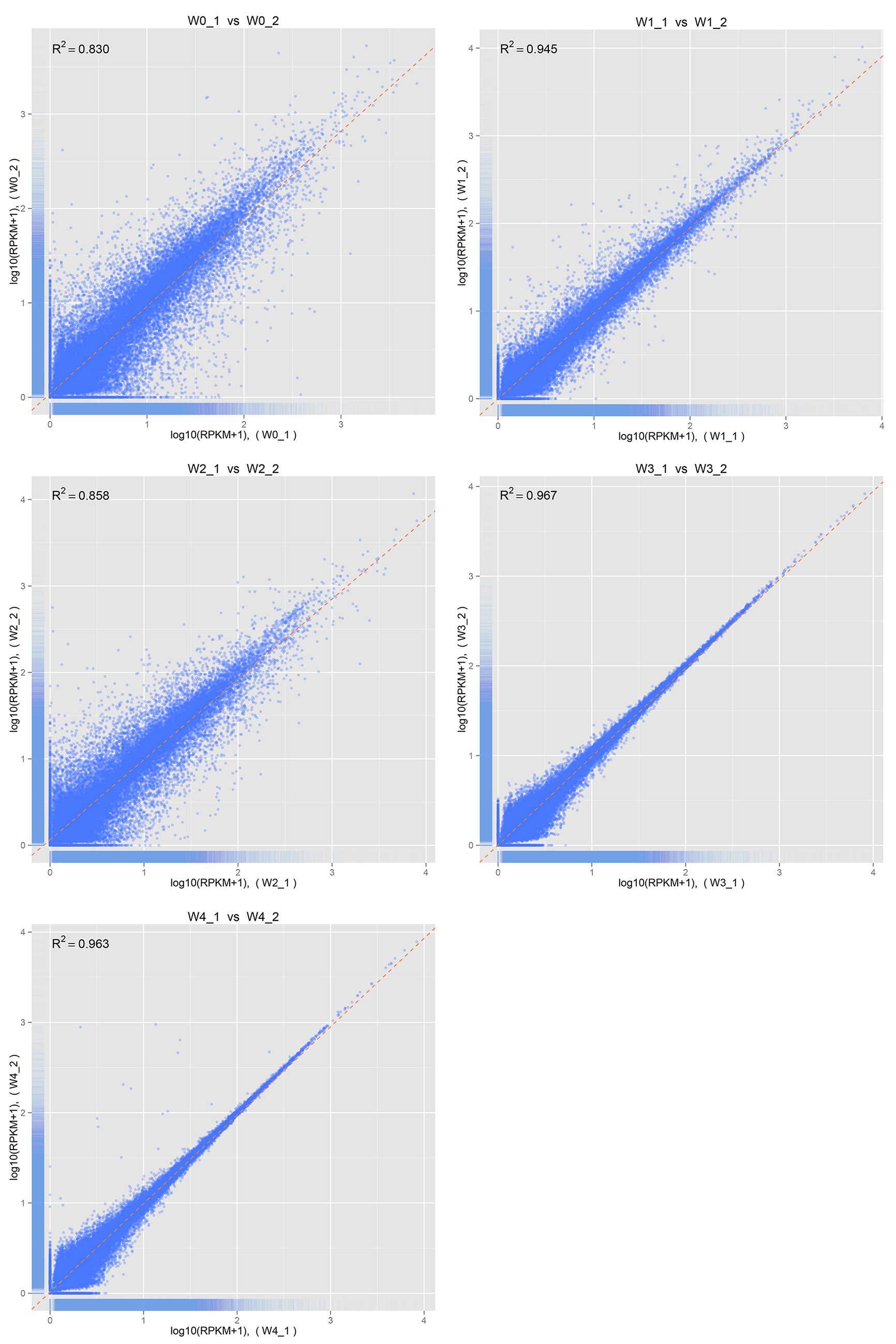

Supplement: Figure S3 — The Pearson correlation coefficients between replicates were calculated by SPSS with transformation of log10(RPKM+1). (TIF) [file pone.0113768.s003.tif]
